# Validity and Reliability of the Portuguese Version of the Healthy Lifestyle Questionnaire—EVS III

**DOI:** 10.3390/ijerph19031612

**Published:** 2022-01-30

**Authors:** Marco Batista, Marta Leyton-Román, Ruth Jiménez-Castuera

**Affiliations:** 1 Department of Sports and Well-Being, Higher School of Education—SHERU (Sport, Health and Exercise Research Unit), Polytechnic Institute of Castelo Branco, Rua Prof. Dr. Faria de Vasconcelos, 6200 Castelo Branco, Portugal; 2 Department of Didactics of Music, Plastic and Body Expression, Faculty of Teaching Training, University of Extremadura. Av. de la Universidad, s/n, 10071 Cáceres, Spain; mleyton@unex.es; 3 Department of Didactics of Music, Plastic and Body Expression, Faculty of Sport Sciences, University of Extremadura, Av. de la Universidad, s/n, 10003 Cáceres, Spain; ruthji@unex.es

**Keywords:** healthy lifestyles, confirmatory factor analysis, motivation, psychometrics, health

## Abstract

The main objective of this study was to validate the Healthy Lifestyle Questionnaire—EVS III, using confirmatory factor analysis of the measurement model. A total of 822 Portuguese individuals of both genders, aged between 18 and 66 years old (*M* = 28.43 *SD* = 12.07), participated in this study, of which 382 were male (46.5%) and 440 were female (53.5%). The main results obtained revealed that the psychometric qualities prove the adequacy of the factor structure of the Healthy Lifestyles Questionnaire—EVS III (7 factors/32 items) and that it has acceptable validity indices: *χ*^2^ = 644.6828, *p* = 0.000, *df* = 168, *χ*^2^/*df* = 3.84, *NFI* = 0.901, *TLI* = 0.902, *CFI* = 0.921, *IFI* = 0.922, *MFI* = 0.900, *GFI* = 0.909, *AGFI* = 0.901, *RMR* = 0.073, *SRMR* = 0.059 and *RMSEA* = 0.059, enabling the assessment of factors related to a balanced diet, respect for mealtimes, tobacco consumption, alcohol consumption, consumption of other drugs, resting habits and physical activity habits. The Portuguese version of the Healthy Lifestyles Questionnaire—EVS III can be used with reasonable confidence for the assessment of healthy lifestyles.

## 1. Introduction

Maintaining a healthy lifestyle is key for managing risk factors for disease and promoting preventive health measures. Examples of this are healthy and health-promoting behaviors, such as good nutrition and weight control, leisure practices, regular physical activity, rest and relaxation periods, the ability to face adverse conditions or situations and to establish supportive, affective relationships and citizenship and adopting an attitude with the objective of living well and with health [1].

Non-health-promoting lifestyle habits are linked to sedentary lifestyles, unbalanced diets, lack of rest, as well as the consumption of harmful substances. In the long term, these lifestyles are associated with diseases such as overweight, type II diabetes, hyper cholesterol and even cancer, leading to a premature increase in morbidity and mortality [2,3]. The importance of adopting a healthy lifestyle from an early age becomes unequivocal [4].

There is a general interest in evaluating and measuring the lifestyles adopted by the general population. Throughout the history of the last 150 years, different instruments have been developed and applied to one or more lifestyle factors, with an impact on different population groups [5].

Upon reviewing the literature on lifestyle assessment, certain behaviors are identified, which call for standardization in this study domain. Habits related to balanced nutrition (balanced eating and respecting mealtimes) [6], physical exercise [7] and resting habits [8] are related to a healthy lifestyle. The unhealthy lifestyle habits identified were a sedentary lifestyle, lack of rest, smoking, the use of other drugs and excessive alcohol intake [9].

In the last two decades, studies that reflect the integration of healthy behaviors in different contexts, or even a different relationship of psychological dimensions with lifestyles, have been highlighted. An example of this is the contrast with the motivation continuum, the motivation forms or the basic psychological needs, seeking to perceive the degree of self-determination for a given practice, for the adoption of healthy behaviors or as a predictor for the abandonment of unhealthy behaviors [5].

Most of the surveys reviewed consider motivation as a key element for achieving adherence to a healthy lifestyle [5]. The Self-Determination Theory [10,11] has been used as a theoretical study model to explain such adherence.

The Self-Determination Theory explains that motivation is presented in a continuum characterized by different levels of self-determination, which, from the highest to the lowest, are intrinsic motivation, extrinsic motivation and amotivation. The authors [10,11] have therefore considered that the most internal dimension of motivation was an autonomous motivation for the involved agent, which derives from interest, satisfaction or which moves actions that are consistent with the being. In turn, the outermost motivation dimension turned this into a controlled motivation, defined by behavior as a function of external contingents, which could create great pressure on individuals to achieve expectations.

Research tends to reveal positive correlations, although not always significant, between autonomous motivation and health-promoting behaviors and negative correlations with behaviors that are harmful to health. Controlled motivation and amotivation have shown negative correlations with health-promoting behaviors and positive correlations with unhealthy behaviors [1,5].

This study aims to validate the Portuguese version of the Healthy Lifestyles Questionnaire—EVS III. The use of this questionnaire will allow the characterization of healthy habits and lifestyles of people in general, regardless of the activity levels or sedentary lifestyle that characterize them. This questionnaire is more comprehensive given the validation of the EVS II carried out by the authors [5] including what has been the future perspective of research, which pointed to a factor inclusion to measure physical activity habits. The Healthy Lifestyles Questionnaire—EVS III presents itself as a new instrument, more adequate in terms of extension and structure, which has an adequate number of items per factor.

## 2. Materials and Methods

### 2.1. Research Design

This study was a cross-sectional correlational study [12]. As for the manipulation of direct interventions on the study object, this was an observational, descriptive study, as there was no manipulation of the independent variables [12].

### 2.2. Participants

The study sample consisted of 822 Portuguese citizens from the general population of mainland Portugal and the islands of Madeira and Azores. Participants were of both genders and aged between 18 and 66 years (*M* = 28.43 *SD* = 12.07). In total, 382 were males (46.5%) and 440 were females (53.5%). Regarding their main activity, the participants were students, active professionals from different national professional framework categories, unemployed and retired. The respondents’ academic qualifications ranged from basic education to doctorate degrees, with the majority having higher education. Concerning the practice of regular physical activity, 220 (26.8%) did not engage in physical activity, 160 (19.5%) reported doing so for less than six months and 442 (53.8%) have been engaging in physical activity for over six months.

The type of sampling used to select the study sample was random [13], since it was not a probabilistic approach, as a participatory approach is inherent to data collection for the population in general.

### 2.3. Instruments

The resulting instrument is called the Healthy Lifestyles Questionnaire (Questionário de Estilos de Vida Saudáveis—EVS III). This questionnaire is more comprehensive than the EVS II validation carried out by Batista et al. [5], including the perspective of future research, which pointed to the inclusion of a factor to measure physical activity habits. As occurred in the validation of the Healthy Lifestyles Questionnaire (EVS II sp) [1], by including a factor to assess healthy lifestyles, we translated these items from Spanish to Portuguese and aggregated them with the EVS II instrument already validated [5].

The EVS III has a 35-item version, using a Likert-type scale that ranges between strongly disagree (1) and strongly agree (5). All questions refer to four domains, namely eating habits, consumption of harmful substances, resting habits and physical activity habits, grouping these into seven factors: Eating habits that include (a) a balanced diet and (b) respecting mealtimes; consumption of harmful substances, which includes (c) tobacco consumption, (d) alcohol consumption and (e) consumption of other drugs; resting habits with a single designated dimension, (f) resting habits; and physical activity habits with a single designated dimension, (g) physical activity habits.

Regarding the measurement of eating habits, it was based on a total of 11 items, specifically, balanced diet (e.g., “I usually eat fish two or more times a week.”)—six items; respecting mealtimes (e.g., “I usually respect mealtimes.”)—five items; the consumption of harmful substances was based on a total of 15 items, tobacco consumption (e.g., “I smoke regularly.”)—five items, alcohol consumption (e.g., “I drink alcoholic beverages regularly on weekends (beer, liquors, wines, combined drinks…).”—five items, consumption of other drugs (e.g., “I’ve never tried drugs (joints, marijuana, cocaine, stimulants,…)”—five items, resting habits (e.g., I normally sleep 7–8 h a day.”)—four items, and physical activity habits (e.g., “I consider myself a physically active person.”)—five items.

To determine the concurrent validity, we used the Behavioral Regulation in Exercise Questionnaire (BREQ3) [14], validated in the Portuguese context [15], which is composed of 24 items, divided into six subscales evaluated according to a five-level Likert-type scale, which varies between zero (not true for me) and four (very often true for me).

These items reflect underlying types of motivation based on the motivational continuum of the Self-Determination Theory, namely amotivation, controlled motivation (external motivation, introjected motivation) and autonomous motivation (identified motivation, integrated motivation and intrinsic motivation). This questionnaire begins with an introductory phrase “Why do you exercise?”, followed by 24 items to measure the different types of motivation, grouped into intrinsic regulation (four items, e.g., “I exercise because it’s fun.”), integrated regulation (four items, e.g., “I exercise because it is related to my life goals.”), identified regulation (four items, e.g., “I value the benefits/advantages of exercise.”), introjected regulation (four items, e.g., “I feel guilty when I don’t exercise.”), external regulation (four items, e.g., “I exercise because other people say I should.”) and amotivation (four items, e.g., “I don’t see why I have to exercise.”).

### 2.4. Institutional Reviewer Board Statement

This study received approval from the Bioethics and Biosafety Commission of the University of Extremadura (Spain), with the registration number R011-0322020, following the guidelines of the Declaration of Helsinki. All participants were treated in accordance with the American Psychological Association ethical guidelines regarding participant consent, confidentiality and anonymity. Written informed consent was obtained from all participants.

### 2.5. Procedures

A back-translation process was performed for items [13] that assessed the physical activity habits subscale of the Healthy Lifestyles Questionnaire (EVS II sp) [1]. Thus, the questionnaire was first translated into Portuguese and later translated again into Spanish by a translator who was external to the research group, who also observed great similarity after the back-translation process. Subsequently, the items were evaluated by three experts in the field [13] who considered that they were adequate for assessing the construct for which they were created. Once the habits and physical activity items were translated, they were integrated into the Healthy Lifestyles Questionnaire—EVS II [5], and this version of the Healthy Lifestyles Questionnaire—EVS III had 35 items.

Once the instrument was created, the questionnaire was applied to a small group of individuals aged 18 years or over, according to the age group intended for the study. Since we targeted the adult population in general, this procedure was aimed at verifying the ability of individuals to understand the questionnaire without signaling any reading comprehension issues.

Subsequently, a dossier was prepared with the different questionnaires to be applied, where we collected data of interest such as gender, age, academic qualifications, occupation, place of residence and regular physical activity habits. Afterwards, the questionnaires were implemented on the Google Forms platform, to be completed online. The administration of the questionnaires was carried out through different channels (WhatsApp, Facebook and email), appealing to the voluntary participation of the population in general. Respondents accessing the online questionnaire had to mark a box to provide their informed consent upon agreement to participate in the study, and they were informed that their anonymity would be respected at all times. None of the respondents received compensation for their participation and could withdraw from the study at any time, simply by sending an email to the person responsible for the study. The data collection period was between March and May 2021. The approximate time estimated for completing the questionnaire was approximately fifteen minutes.

### 2.6. Data Analysis

The statistical data analysis was performed using SPSS statistical software (version 23.0 for Windows, SPSS, Inc., Chicago, IL, USA). Initially, we proceeded to filter the data in order to see if there was any missing data. Since there were no missing data, we analyzed the data normality. For the univariate normality analysis, the asymmetry and kurtosis indicators [16] were used for each item that makes up the EVS III. The proposed values were then considered normal (up to 2 for asymmetry and 7 for kurtosis), moderately normal (between 2 and 3 for asymmetry and between 7 and 21 for kurtosis), or extremely normal (values greater than 7 for asymmetry and 21 for kurtosis).

We estimated the construct validity, respecting the elimination criterion of items whose regression weight did not present an adequate value (less than 0.40), and the factor loadings of each item should be significant [17].

To verify whether the number of factors was reasonable based on the specific measurement model presented, we performed the calculation of the Omega hierarchical subscale coefficient (OmegaHS) proposed by the authors [18]. The OmegaHS calculation can be considered an indicator of latent variable strength specific to the factors that constitute a variable. The authors [18] point out that values close to 0.00 are indicative of a very weak specific latent variable, whereas values close to 1.0 are indicative of a very strong specific latent variable, categorizing them as follows: Very small <0.10; relatively small <0.20; 0.20 to 0.30 typical; and relatively large >0.30.

Confirmatory factor analysis (CFA) was performed using EQS software (version 6.1 for Windows, Multivariate Software, Inc., Los Angeles, IL, USA). To test the adequacy of the structural equation model, the maximum likelihood (ML) method was used. For this purpose, we used the following recommended indicators [19]: *χ*^2^, *χ*^2^/*df*, *NFI* (Normed Fit Index), *TLI* (Tucker Lewis Index), *CFI* (Comparative Fit Index), *GFI* (Goodness of Fit Index), *AGFI* (Adjusted Goodness of Fit Index), *RMSEA* (Root Mean Square of Approximation) and *SRMR* (Standardized Root Mean Square Residual).

The determination of *χ*^2^ indicates a similarity of the observed covariates with those predicted in the hypothetical model, using the following reference values for a good fit: 0 ≤ *χ*^2^ ≤ 2*df* and an acceptable fit as 2*df* < 2 ≤ 3*df*. However, as *χ*^2^ is very sensitive to the sample size, it is recommended to complete this with *χ*^2^/*df*, for which values below 2 indicate a very good model fit, with values below 3 being considered acceptable [19].

The incremental indices (*NFI*, *TLI*, *CFI*, *GFI* and *AGFI*) compare the hypothetical model with the null model and are not affected by the sample size. We considered reference values as acceptable when they were higher than 0.90 and good when they were higher than 0.95 [19].

The same authors [19] propose that the values of the error rates *RMSEA* and *SRMR* be lower than 0.08 for an acceptable fit and lower than 0.05 for a good fit, and the respective standardized factor loadings should all be statistically significant (*p* < 0.01).

As a criterion for model eligibility, we met the normalized Mardia coefficient. Compliance with values below 5 of this criterion is interpreted as a normal distribution and allows us to use the maximum likelihood method [19]. When we do not obtain a normal multivariate distribution, we apply the *χ*^2^ statistical corrective robustness measure [20].

A descriptive analysis was carried out by the determination of the mean and standard deviations of each extracted factor, and the concurrent validity was evaluated through a bivariate correlation analysis. This concurrent validity assessment is justified because, according to the theoretical conceptual framework of the Self-Determination Theory [10,11], autonomous motivation appears positively correlated with healthy behaviors and negatively correlated with unhealthy behaviors. Instead of this logic, it is natural for controlled motivation and motivation to appear negatively correlated with healthy behaviors and positively with non-healthy behaviors [21,22]. The interpretation of the correlation direction was based on the positive or negative sign of the correlation coefficient r. Considering the proposed values [13], we classified values above 0.9 as a very strong correlation, 0.7 to 0.9 as a strong correlation, 0.5 to 0.7 as a moderate correlation, 0.3 to 0.5 as a weak correlation and 0 to 0.3 as a minimal correlation.

## 3. Results

### 3.1. Descriptive Statistics and Reliability

According to the descriptive perspective (Table 1), values consistent with a healthy lifestyle were obtained, with higher means in balanced diet behaviors (*x*^−^ = 3.55 ± 0.94), respecting mealtimes (*x*^−^ = 3.51 ± 0.97), resting habits (*x*^−^ = 3.46 ± 1.04) and physical activity habits (*x*^−^ = 3.33 ± 1.07), and lower means of tobacco consumption (*x*^−^ = 1.49 ± 0.90), alcohol consumption (*x*^−^ = 1.60 ± 0.78) and consumption of other drugs (*x*^−^ = 1.53 ± 0.73).

In the present work, we have chosen to eliminate three items, since they did not meet the factorial load equal to or greater than 0.40, as proposed by the author [17].

The internal consistency of each of the factors resulting from the factor analysis (Cronbach’s alpha) presented the following results: (0.77) balanced diet, (0.81) respecting mealtimes, (0.89) tobacco consumption, (0.84) alcohol consumption, (0.74) consumption of other drugs, (0.81) resting habits and (0.86) physical activity habits. McDonald’s Omega (ω) coefficient, as a more robust complement to the previous indicator, revealed the following results: (0.84) balanced diet, (0.86) respecting mealtimes, (0.94) tobacco consumption, (0.88) alcohol consumption, (0.84) consumption of other drugs, (0.84) resting habits and (0.92) physical activity habits.

The average variance extracted and the composite reliability for each factor were 0.58 and 0.85 for balanced nutrition, 0.61 and 0.86 for respecting mealtimes, 0.78 and 0.94 for tobacco consumption, 0.64 and 0.88 for alcohol consumption, 0.57 and 0.84 for consumption of other drugs, 0.57 and 0.84 for resting habits and 0.75 and 0.92 for physical activity habits, fulfilling all the evaluated factors, as proposed by the author [23].

In most factors, the Omega HS values showed a relatively large (>0.30) specific latent variable strength, with only the factor for the consumption of other drugs showing a typical value (0.20 to 0.30).

### 3.2. Confirmatory Factor Analysis

A confirmatory factor analysis to assess the seven-factor model of the Healthy Lifestyles Questionnaire—EVS III version showed that the 32 items were grouped into seven factors: Balanced diet (four items), respecting mealtimes (five items), tobacco consumption (five items), alcohol consumption (five items), consumption of other drugs (five items), resting habits (three items) and physical activity habits (five items).

Table 2 shows three measurement models were tested based on the loading factor of each item in the respective factor. There was a need to perform this procedure because the incremental indices of the 32-item model did not meet theoretical assumptions proposed for measurement models in confirmatory factor analysis. Model one was conceived by framing four items per factor, and model two and model three integrated three items per factor.

Table 3 shows that the adjustment indicators that best comply are those of model three. Models one and two, despite presenting most of the adjustment indicators for the respective acceptable measurement models, did not fully comply with them, namely model one in the incremental indices *MFI*, *GFI*, *AGFI* and *SMR*, as well as model two in the incremental indices *MFI*, *AGFI* and *SMR*.

**Figure 1:**
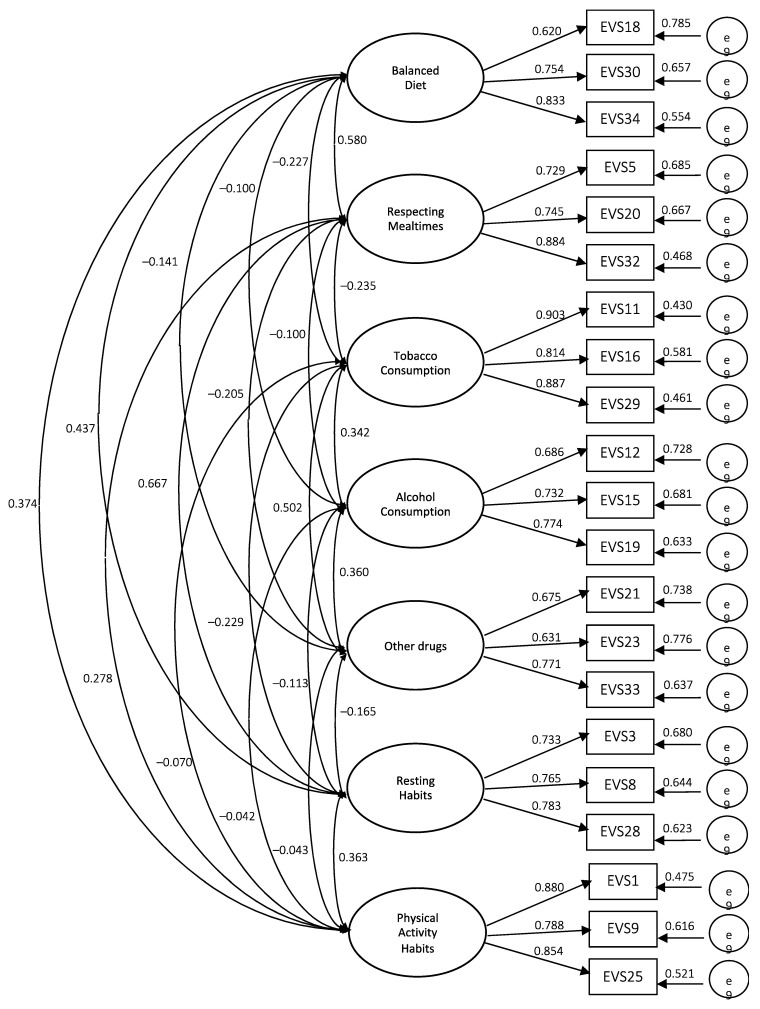
Structural model of the Healthy Lifestyles Questionnaire—EVS III.

In all models tested, the standardized loading factors were all statistically significant (*p* < 0.01), thus we can conclude that at the analytical level, the results presented by model three are more satisfactory (Figure 1).

After a detailed analysis of the global results (Table 4), the third model that was tested indicated a reasonable fit of the Healthy Lifestyle Questionnaire—EVS III: *χ*^2^ = 644.6828, *p* = 0.000, *df* = 168, *χ*^2^/*df* = 3.84, *NFI* = 0.901, *TLI* = 0.902, *CFI* = 0.921, *IFI* = 0.922, *MFI* = 0.900, *GFI* = 0.909, *AGFI* = 0.901, *RMR* = 0.073, *SRMR* = 0.059, *RMSEA* = 0.059. With these results, structural model three revealed an acceptable global fit, just as models with satisfactory fit had been obtained in previous versions, although with fewer analysis dimensions than the Healthy Lifestyles Questionnaire—EVS III.

### 3.3. Validity (Concurrent Validity Analysis)

According to the descriptive perspective, regarding motivation variables, respondents demonstrated high autonomous motivation (*x*^−^ = 2.82 ± 0.94), and low controlled motivation (*x*^−^ = 1.05 ± 0.63) and amotivation values (*x*^−^ = 0.45 ± 0.71).

The concurrent validity assessment was performed using a bivariate correlation analysis, revealing that most of the correlations between the EVS III and BREQ 3 variables were significant and in the expected sense. Autonomous motivation showed minimal positive correlations with a balanced diet, respecting mealtimes and resting habits, and a moderate positive correlation with physical activity habits. It also assumed a minimal positive correlation with controlled motivation and a weak negative correlation with amotivation, and minimal negative correlations with tobacco consumption, alcohol consumption and consumption of other drugs. Controlled motivation showed minimal negative correlations with eating habits, and minimal positive correlations with resting and physical activity habits, as well as with lifestyle variables that are harmful to health. Amotivation presented minimal negative correlations with the various healthy lifestyle variables and minimal positive correlations with controlled motivation and the unhealthy lifestyle variables.

## 4. Discussion

The main objective of this study was to follow the recommendations of previous research on healthy lifestyles in a Portuguese population [5], via the validation of the new and more complete Healthy Lifestyles Questionnaire—EVS III for the Portuguese population in general.

Considering that a new application of a measurement instrument represents a contribution to improving the theoretical research domain value [16], this study expands the body of knowledge regarding lifestyles in Portugal. By confirming the validity of the Healthy Lifestyles Questionnaire—EVS III, this instrument is available for further research, allowing the expansion of knowledge and healthy practices, as the health indicators of the population.

The loading factor, extracted by the correlation between the item and factor, was equal to or greater than 0.5 (FL > 0.5), as recommended [18]. The same author also indicated that factorial weights above 0.70 are considered indicative of a very well-defined structure, with least 50% explained by the factor of the item variance [18], which were the factorial weights that we obtained in most items.

After estimating the composite reliability and the average variance extracted for each factor, we observed that the obtained values respect the proposed indicators [18], to conclude that a substantial amount of the variance is captured by the construct, where the composite reliability must have a minimum value of 0.70, and the average variance extracted is greater than 0.50.

The OmegaHS calculation allowed us to verify whether the number of factors is reasonable considering the specific measurement model presented, obtaining a relatively large latent force in each factor (>0.30), as ideally intended [18].

The internal consistency indicators used, i.e., Cronbach’s alpha and McDonald’s omega calculated for each of the factors, showed values greater than or equal to 0.70, as proposed in the literature [18].

Confirmatory factor analysis showed that the 32 items were grouped into seven factors: Balanced diet (four items), respecting mealtimes (five items), tobacco consumption (five items), alcohol consumption (five items), consumption of other drugs (five items), resting habits (three items) and physical activity habits (five items). Of the three models tested, structural model three revealed a satisfactory global fit, as well as models with a satisfactory fit obtained in previous versions, although with a smaller number of factors in the analysis than the Healthy Lifestyles Questionnaire—EVS III.

The results obtained in the psychometric quality indices [19] revealed an acceptable fit in *χ*^2^, and in the values of *χ*^2^/*df*, *NFI*, *GFI* and *RMSEA*. The results also showed a good fit in the *AGFI* and *SRMR* indices. Despite the values of TLI and CFI being very close to those indicated [18], they did not comply with the values proposed by some authors [28,29]. These results are in line with previous research using the Healthy Lifestyle Questionnaire [1,5,24,25,26,27] and highlight the importance of each of the seven dimensions in understanding and studying healthy lifestyles. If we detail the results of the research that used the Healthy Lifestyle Questionnaire [1,5,24,25,26,27] with those obtained in our study, they all present adequate psychometric properties [18,28,29], highlighting the Healthy Lifestyle Questionnaire in the six published studies and in the present study as well as an adequate and reliable instrument for the assessment of lifestyles.

We emphasize that the Healthy Lifestyle Questionnaire—EVS III validation is one of the most complete versions, which best respects the initial questionnaire model presented by Wold [30] with seven extracted factors, similar to what the authors [1] obtained in the EVS II sp or what Batista et al. [5] validated with six factors.

The previously validated versions revealed problems with some items with a loading factor less than 0.40 [17] leading to the elimination of some of the extracted factors, which was the case of resting habits [24], or the items agglutination from a balanced diet and respecting mealtimes factors, which gave rise to the dietary habits factor [25]. In the present study, out of the 35 items, we also chose to eliminate 3 items, as they did not meet a loading factor equal to or greater than 0.40 [17], specifically two items in the balanced diet factor and one in the resting habits factor. The instrument has the potential to become more refined, as new contributions to healthy lifestyles emerge, in which case it is recommended for future studies to remeasure and test these items to obtain other valid equation models [17,18].

The values obtained in the descriptive analysis showed moderate and high means in the dimensions of a balanced diet, respecting mealtimes, resting habits and physical activity habits. At the same time, there were reduced means in the dimensions of tobacco consumption, alcohol consumption and consumption of other drugs, showing the theoretical importance underlying the construct of healthy lifestyles. These descriptive trends were also observed in various studies underlying the use of the Healthy Lifestyles Questionnaire [1,5,24,25,26,27].

Regarding concurrent validity, the bivariate correlation analysis between the variables that constitute the EVS III and BREQ 3 showed significant associations in the expected sense, evidencing adequate concurrent validity tested in this work, mainly with the motivation continuum proposed by the Theory of Self-determination [10,11]. This trend corroborates the concurrent validity trend observed in previous instrument validations [1,5,24,25,26,27]. Taking as a reference a previous meta-analysis investigation [31] including a large sample of non-experimental studies, it was determined that there is a strong relationship between the Self-Determination Theory assumptions [10,11] and behavior adoption or maintenance that promote health. Overall, it can be considered that the present version of the Healthy Lifestyles Questionnaire—EVS III presents adequate concurrent validity, considering the Self-Determination Theory [10,11] theoretical framework.

Despite the obtained values that will allow validation of the EVS III Questionnaire for a population with a wide age range, some limitations must be considered. The questionnaire was applied to a large sample; however, in the future and following the authors’ recommendations [1], it could be applied to groups of people with specific pathologies such as cancer, hypertension and obesity, in order to determine the validity of the questionnaire in these groups. It could also be a useful tool to determine the lifestyle carried out by these groups once an intervention program has begun, after a surgical operation or even to determine the initial patient diagnosis.

It would also be interesting to predict other psychological variables based on EVS III factors (e.g., emotions, depression, etc.), which would help to generate strategies that can help people to lead healthier lifestyles. Another possible limitation is related to the variance in areas and participants’ ages, for example those who come from Continental (mainland) Portugal and those who come from the islands, and the differences that this may imply. Therefore, it would be interesting to determine the psychometric properties of the questionnaire in specific areas, genders and ages, as well as in other countries or cultural contexts. An EVS III questionnaire expansion with a factor inclusion that assesses sedentary behavior may be equally pertinent.

Other perspectives are the development of studies that are based on the transtheoretical motivation model [32], or on the planned behavior theory [33] or even others that are also based on the Self-Determination Theory [10,11] and that consequently assess the adoption of healthy lifestyles in various strata of the population.

## 5. Conclusions

In conclusion, the adaptation of the Portuguese version of the Healthy Lifestyles Questionnaire—EVS III, with seven factors, can be used for the assessment of healthy lifestyles, underlying eating habits, consumption of harmful substances, resting habits and physical activity habits.

The results indicate that both the factorial and reliability validity of the Portuguese version of the Healthy Lifestyles Questionnaire—EVS III are acceptable for the general population aged between 18 and 66 years.

## Figures and Tables

**Table 1:** Descriptive statistics, internal consistency values, discriminant and convergent validity of EVS III.

Variable	Item	*M*	*SD*	*FL*	*CF*	*Skew*	*Kurt*	*OHS*	*α*	*Ω*	*VME*	*FC*
Balanced Diet	EVS 10	3.55	0.94	0.725	0.668 *	−0.40	−0.46	0.34	0.77	0.84	0.58	0.85
	EVS 18			0.698	0.725 *							
	EVS 30			0.807	0.787 *							
	EVS 34			0.843	0.853 *							
Respecting Mealtimes	EVS 5	3.51	0.97	0.723	0.750 *	−0.47	−0.41	0.39	0.81	0.86	0.61	0.86
	EVS 20			0.774	0.792 *							
	EVS 24			0.736	0.700 *							
	EVS 31			0.696	0.660 *							
	EVS 32			0.853	0.879 *							
Tobacco Consumption	EVS 2	1.49	0.90	0.860	0.851 *	1.76	1.90	0.47	0.89	0.94	0.78	0.94
	EVS 6			0.590	0.628 *							
	EVS 11			0.916	0.907 *							
	EVS 16			0.864	0.877 *							
	EVS 29			0.919	0.904 *							
Alcohol Consumption	EVS 7	1.60	0.78	0.767	0.786 *	1.35	1.10	0.33	0.84	0.88	0.64	0.88
	EVS 12			0.846	0.808 *							
	EVS 13			0.796	0.766 *							
	EVS 15			0.779	0.815 *							
	EVS 19			0.762	0.790 *							
Consumption of Other Drugs	EVS 4	1.53	0.76	0.448	0.548 *	1.54	1.66	0.30	0.74	0.84	0.57	0.84
	EVS 21			0.751	0.717 *							
	EVS 23			0.712	0.785 *							
	EVS 27			0.799	0.716 *							
	EVS 33			0.789	0.798 *							
Resting Habits	EVS 3	3.46	1.04	0.854	0.857 *	−0.40	−0.61	0.36	0.81	0.84	0.57	0.84
	EVS 8			0.874	0.871 *							
	EVS 28			0.819	0.817 *							
Physical Activity Habits	EVS 1	3.33	1.07	0.872	0.876 *	0.05	−1.10	0.44	0.86	0.92	0.75	0.92
	EVS 9			0.852	0.860 *							
	EVS 17			0.842	0.841 *							
	EVS 25			0.874	0.874 *							
	EVS 35			0.508	0.491 *							

*M*—Mean; *SD*—Standard Deviation; *FL*—Factor Loading: Correlation between item and factor; *CF*—Factor loading of the item in the factor * *p* < 0.01; *Skew*—Skewness; *Kurt*—Kurtosis; *OHS*—Omega hierarchical subscale coefficient; *α—* Cronbach’s Alpha; *Ω*—McDonald’s Omega; VME—Average variance extracted; *FC*—Composite reliability.

**Table 2:** Fit indices of the three measurement models tested for the Healthy Lifestyles Questionnaire—EVS III.

	Model 1	Model 2	Model 3
*X* ^2^ *Sig X* ^2^	1058.0387 (*p* < 0.001)	676.5737 (*p* < 0.001)	644.6828 (*p* < 0.001)
*df*	303	168	168
*X*^2^/*df*	3.49	4.02	3.84
*NFI*	0.884	0.903	0.901
*TLI*	0.900	0.906	0.902
*CFI*	0.914	0.925	0.921
*IFI*	0.914	0.925	0.922
*MFI*	0.734	0.869	0.900
*GFI*	0.849	0.900	0.909
*AGFI*	0.879	0.863	0.901
*RMR*	0.084	0.082	0.073
*SRMR*	0.058	0.059	0.059
*RMSEA*	0.055	0.061	0.059
90% CI *RMSEA*	(0.066–0.073)	(0.056–0.065)	(0.054–0.064)

**Table 3:** Fit indices model for the Healthy Lifestyle Questionnaire (EVS).

	EVS vp	EVS	EVS sp	EVS II	EVS spII	EVS eq	EVS III
*X*^2^ Sig *X*^2^	632.68 (*p* < 0.001)	172.117 (*p* < 0.001)	- (*p* < 0.001)	305.925 (*p* < 0.001)			644.6828 (*p* < 0.001)
*df*	157.775	41.078	-	120.017			168
*X*^2^/*df*	4.010	4.190	4.2	2.549	3.76	9.02	3.84
*NFI*	-	0.956	-	0.909			0.901
*TLI*	-	0.955	-	0.918			0.902
*CFI*	0.940	0.966	0.940	0.944	0.973	0.96	0.921
*IFI*	0.940	0.966	0.940	0.946		0.96	0.922
*MFI*	-	0.909	-	0.901		0.91	0.900
*GFI*	0.920	0.955	-	0.944			0.909
*AGFI*	-	0.927	-	0.909		0.94	0.901
*RMR*	-	0.049	-	0.051			0.073
*SRMR*	0.060	0.043	0.040	0.048	0.059	0.03	0.059
*RMSEA*	0.070	0.068	0.060	0.060	0.049	0.06	0.059
90% CI *RMSEA*	-	(0.058–0.076)	-	(0.056–0.072)	(0.046–0.052)		(0.054–0.064)

EVS vp—Portuguese preliminary version [24]; EVS—Portuguese version [25]; EVS sp—Castilian version [26]; EVS II—Portuguese version [5]; EVS II sp—Castilian version [1]; EVS eq—Ecuadorian version [27]; EVS III—Present version of the Portuguese validation.

**Table 4:** Bivariate correlation between EVS III and BREQ 3 variables.

Variable	Mean	*SD*	1	2	3	4	5	6	7	8	9	10
1. Balanced Diet	3.55	0.94	-	0.57 **	−0.25 **	−0.07	−0.14 **	0.41 **	0.43 **	0.28 **	−0.06	−0.15 **
2. Respecting Mealtimes	3.51	0.97		-	−0.28 **	−0.12 **	−0.22 **	0.55 **	0.34 **	0.24 **	−0.03	−0.11 **
3. Tobacco Consumption	1.49	0.90			-	0.33 **	0.53 **	−0.20 **	−0.09 **	−0.08 *	0.08 *	0.12 **
4. Alcohol Consumption	1.60	0.78				-	0.37 **	−0.07 *	0.02	−0.03	0.04	0.10 **
5. Other Drugs	1.53	0.76					-	−0.14 *	0.05	−0.06	0.17 **	0.01
6. Resting Habits	3.46	1.04						-	0.34 **	0.25 **	0.01	−0.13 **
7. Physical Activity Habits	3.33	1.07							-	0.67 **	0.11 **	−0.30 **
8. Autonomous Motivation	2.82	0.94								-	0.24 **	−0.42 **
9. Controlled Motivation	1.05	0.63									.	0.30 **
10. Ammotivation	0.45	0.71										-

Note: * *p* < 0.05; ** *p* < 0.01.

## Data Availability

The data presented in this study are available by request from the corresponding author. The data are not publicly available due to ethical and private reasons.
